# Dose-dependent stimulation of human follicular steroidogenesis by a novel rhCG during ovarian stimulation with fixed rFSH dosing

**DOI:** 10.3389/fendo.2022.1004596

**Published:** 2022-10-20

**Authors:** Jane Alrø Bøtkjær, Stine Gry Kristensen, Hanna Ørnes Olesen, Per Larsson, Bernadette Mannaerts, Claus Yding Andersen

**Affiliations:** ^1^ Laboratory of Reproductive Biology, University Hospital of Copenhagen, Rigshospitalet, Copenhagen, Denmark; ^2^ Global Biometrics, Ferring Pharmaceuticals A/S, Copenhagen, Denmark; ^3^ Reproductive Medicine & Maternal Health, Ferring Pharmaceuticals A/S, Copenhagen, Denmark; ^4^ Faculty of Health and Medical Sciences, University of Copenhagen, Copenhagen, Denmark

**Keywords:** Steroidogenesis, FE 999302, recombinant hCG, ovarian stimulation, cumulus cells, single nucleotide polymorphisms

## Abstract

**Background:**

Choriogonadotropin (CG) beta (FE 999302), a novel recombinant human (h)CG produced by a human cell line, has a longer half-life and higher potency than CG alfa produced by a Chinese hamster ovary cell line. hCG augments steroid production, but the extent of which CG beta treatment during ovarian stimulation (OS) increases steroidogenesis is unknown.

**Objective:**

To explore how increasing doses of CG beta during OS augment follicular steroidogenesis and change gene expression in cumulus cells.

**Study design:**

This study is part of a randomized, double-blind, placebo-controlled trial to investigate the efficacy and safety of CG beta plus recombinant follicle-stimulating hormone (rFSH) in women undergoing OS during a long gonadotrophin-releasing hormone agonist protocol. The study primary endpoint was intrafollicular steroid concentrations after CG beta administration. Secondary outcomes were gene expression of *FSHR* , *LHR*, *CYP19a1*, and *androgen receptor* (*AR*).

**Participants/methods:**

619 women with anti-Müllerian hormone levels 5–35 pmol/L were randomized to receive placebo or 1, 2, 4, 8, or 12 µg/day CG beta from Day 1 of OS plus rFSH. Follicular fluid (FF) (n=558), granulosa (n=498) and cumulus cells (n=368) were collected at oocyte retrieval. Steroid FF hormones were measured using enzyme-linked immunosorbent assays, gene expression was analyzed in cumulus cells by quantitative reverse transcriptase polymerase chain reaction (qRT-PCR) and single nucleotide polymorphism (SNP) analysis was performed in granulosa cells.

**Results:**

17-OH-progesterone, androstenedione, testosterone, and estradiol concentrations significantly increased in a CG-beta dose-dependent manner during OS (p<0.0001), reaching up to 10 times higher values in the highest dose group versus placebo. There was no difference between CG beta dose groups and placebo for progesterone. Expression levels of *CYP19a1* increased significantly in the highest dose group of CG beta (p=0.0325) but levels of *FSHR* , *LHR* and *AR* were not affected by CG beta administration. There were no differences between the *FSHR* (307) or *LHR*(312) SNP genotypes for dose-dependent effects of CG beta in relation to number of oocytes, intrafollicular steroid hormone levels, or gene expression levels.

**Conclusions:**

These results reflect the importance of the combined effect of FSH and hCG/LH during OS on granulosa cell activity, follicle health and potentially oocyte quality.

**Trial Registration number:**

2017-003810-13 (EudraCT Number).

**Trial Registration date:**

21 May 2018.

**Date of first patient’s enrolment:**

13 June 2018. Presented at the 38th Annual Meeting of the European Society of Human Reproduction and Embryology, P-567, 2022.

## Introduction

During human folliculogenesis, granulosa cells (GCs) start to develop luteinizing hormone/human chorionic gonadotrophin receptors (*LHCGR*) around the same time as selection for dominance at a follicle with a diameter greater than 6–8 mm (1). The expression level of *LHCGR* increases and peaks in GCs from preovulatory follicles prior to the final maturation of oocytes and then decreases during the mid-cycle surge of gonadotropins until shortly before ovulation (1). The expression of *LHCGR* is thus highly dynamic and ensures that the selected follicles continue growth and development in an environment of declining follicle-stimulating hormone (FSH) levels as observed in the natural menstrual cycle.

Although both FSH receptors (*FSHR*s) and *LHCGR*s are classical G protein-coupled receptors and activation of these receptors exerts a myriad of known effects on GC function, it is still not clarified to what extent ovarian steroidogenesis, which is especially active during the second half of the follicular phase of the treatment cycle, is stimulated by FSH and/or luteinizing hormone (LH) bioactivity. Whereas FSH levels are decreasing towards ovulation, expression of both *LHCGR* and *FSHR* in GCs of preovulatory follicles points towards a combined effect of LH and FSH in the late follicular phase (1).

Ovarian stimulation (OS) with exogenous gonadotropins is characterized by high and continuous supraphysiological concentrations of FSH, which also results in an upregulation of *LHCGR* on GCs and a higher sensitivity towards LH (2). However, similar to the natural cycle, secretion of estradiol from the mature follicles requires the combined action of theca cells to provide androgen substrate, which becomes aromatized in the GCs, thus, the interplay between theca cells and GCs finally determines the total amount of estrogen produced during OS (3–5).

In connection with OS, exogenous preparations of gonadotropins are normally administered to stimulate multiple follicle development, but there is little consensus on the optimal ratios of FSH and LH activity regarding their actual contributions to steroidogenesis in these follicles. Furthermore, several studies have shown that both endogenous and exogenous administered gonadotropins are active in stimulating steroidogenesis (6). In gonadotropin-deficient women, FSH can stimulate follicular development but in the absence of LH activity, estradiol secretion is minimal, resulting in deficient endometrial growth (7). In addition, LH activity promotes estradiol secretion in a dose-dependent manner and enhances the effect that FSH has on follicular growth in WHO group I and II anovulatory patients (8). In the long gonadotrophin-releasing hormone (GnRH) agonist protocol, the mode of GnRH agonist administration affects circulating levels of endogenous LH and subcutaneous injections of GnRH agonists provide a stronger downregulation as compared with, for instance, intranasal administration (6, 9). Following profound suppression of endogenous gonadotropins in a long GnRH agonist protocol, exogenous gonadotropin preparations containing LH-like activity (i.e., human choriogonadotropin [hCG]) result in higher estradiol production as measured during stimulation at the mid-follicular phase and at the end of stimulation prior to induction of final maturation of oocytes (6, 10).

LH shares the same alpha subunit with hCG and 85% of their amino acid sequence of the beta subunit is also identical (11–13). Both hormones bind and activate the *LHCGR*; however, they have different glycosylation patterns which appear to activate different intracellular signaling cascades leading to a higher potency of hCG compared with LH as well as a longer half-life (14–16). Urinary or recombinant hCG (rhCG) is utilized in assisted reproduction to trigger final follicle maturation and thus replace the naturally occurring LH surge. CG beta is a newly developed rhCG produced by a human cell line (PER.C6^®^) (17). This variant contains the same amino acid sequence as the endogenous hCG but presents a different glycosylation profile compared with urinary hCG or rhCG derived from a CHO cell line. CG beta has also been shown to have a longer half-life and therefore a higher potency (17).

This study is a part of a randomized, double-blind, placebo-controlled trial (the Rainbow trial) that investigated the efficacy and safety of CG beta as an add-on treatment to recombinant FSH (rFSH) in women undergoing OS as well as the effect of CG beta on the number of good-quality blastocysts (18). The trial demonstrated that treatment with CG beta did not affect the duration of stimulation but did reduce the number of intermediate follicles and related downstream parameters including the number of oocytes and blastocysts. Our present study aims to investigate how increasing doses of CG beta combined with an individualized, fixed daily rFSH dose regimen for OS affects follicular steroidogenesis and gene expression in cumulus cells collected at oocyte retrieval from a single large preovulatory follicle in each patient. As previous studies have suggested that common single nucleotide polymorphisms (SNPs) of both *FSHR* and *LHCGR* influence their signaling (19–22), this study further explored two SNPs in the *FSHR* and *LHR* genes, namely the rs2293275 located in exon 10 of the *LHCGR* gene, which causes an amino acid change (asparagine to serine) at residue 312 (N312S), and rs6165 which locates in exon 10 of the *FSHR* gene that results in an amino acid change (alanine to threonine) at residue 307 (A307T). In the current study it was evaluated whether these SNPs impacted the effects of CG beta on the number of oocytes retrieved in connection with OS and the intrafollicular steroid hormone levels.

## Materials and methods

### Study design

This study is a part of a multicenter, double-blind, randomized dose-range trial to investigate the efficacy and safety of CG beta as an add-on treatment to follitropin delta (rFSH, Rekovelle®,Ferring Pharmaceuticals, Denmark) in women undergoing OS with a long GnRH agonist protocol of daily 0.1 mg triptorelin subcutaneously starting in the mid-luteal phase (18). Participants were randomized to receive a daily dose of 1, 2, 4, 8, or 12 μg of CG beta (FE 999302) or placebo along with a fixed daily dose of rFSH individualized according to each participant’s body weight and anti-Müllerian hormone (AMH) levels (Elecsys AMH Plus Immunoassay). Only patients with AMH levels between 5 and 35 pmol/l were included in the trial that applied a long GnRH agonist protocol. The dosing range of CG beta was designed to cover increasing amounts of LH activity and based on the Phase 1 cross-over data in men (17), the lowest dose of 1 µg was estimated to be equi-effective to the hCG dose given during OS with a daily dose of 225 IU menotropin, the latter providing a steady-state hCG level of about 3 IU/L (10).

Final follicular maturation was induced by a single dose of choriogonadotropin alfa 250 µg (rhCG, Ovitrelle®, Merck, USA) if there were <25 follicles with a diameter of ≥ 12 mm and oocyte retrieval was performed 36 h ( ± 2 h) thereafter.

The trial was conducted in 5 countries (Denmark, Belgium, Czech Republic, Spain, and United Kingdom). The trial protocol was approved by the local regulatory authorities and the independent ethics committees covering all participating centers. The trial was started in May 2018 and completed in January 2020 and was performed in accordance with the principles of the Declaration of Helsinki, the International Conference on Harmonization Guidelines for Good Clinical Practice, and local regulatory requirements. All participants provided written informed consent.

### Study participants

A total of 619 women (age 30–42 years) who were undergoing their first or second IVF/ICSI cycle due to unexplained infertility, tubal infertility, endometriosis stage I/II, or with partners diagnosed with male factor infertility, were randomized, and treated in the trial (18). Additional main inclusion criteria were BMI of 17.5–32.0, regular menstrual cycles of 24–35 days, and AMH levels at screening of 5.0–35.0 pmol/L. The main exclusion criteria were poor or excessive ovarian response in a previous OS cycle, endometriosis stage III or IV, history of recurrent miscarriage, and use of hormonal preparations (except for thyroid medication) during the last menstrual cycle before randomization. The patient flow of study participants has previously been reported (18).

The pharmacodynamics of CG beta in combination with rFSH during ovarian stimulation, including serum hormone assays have been described previously (18).

### Steroid hormone concentrations in follicular fluid

At the time of oocyte retrieval, the follicular fluid (FF) (n=558), cumulus cells (n=368), and GCs (n=498) from the first large (≥17 mm) follicle punctured from either ovary was aspirated and stored at –80°C until further analysis. The FF was analyzed for steroid hormone concentrations of progesterone, 17-OH-progesterone, androstenedione, testosterone, and estradiol using commercially available enzyme-linked immunosorbent assays (ELISA). ELISA kits used to detect estradiol, androstenedione, testosterone, and progesterone were purchased from NOVATEC Immundiagnostica Gmbh (www.NovaTec-ID.com, Product no: DNOV003; DNOV008; DNOV002; DNOV006), while the ELISA kit used to detect 17-OH-Progesterone was purchased from IBL International Gmbh (Catalog # RE52071, RRID: AB_2909524). FFs were diluted prior to analysis in 1% bovine serum albumen in 1x phosphate buffered saline for the following steroids: 1:2000 for progesterone, 1:1000 for 17-OH-progesterone, 1:10 for androstenedione, 1:10 for testosterone, and 1:2000 for estradiol.

The ELISA assays used in this study were all validated on different parameters i.e., intra-, and inter-assay variability and freezing time of a given sample pipetted into individual tubes to avoid repeated freeze/thawing steps. The intra-assay variations for the estradiol ELISA assay ranged from 2.0 to 4.2% and the inter-assay variability showed a range of 7.7 to 8.1% for FF samples diluted 1:2000 during measurement. The intra-assay variations for the androstenedione ELISA assay ranged from 2.8 to 6.5% and the inter-assay variability showed a range of 9.4 to 12.3% for FF samples diluted 1:10. The data on the intra-assay variations for the testosterone ELISA assay was in the range from 1.6 to 8.6% and the inter-assay variability showed a range of 3.8 to 7.8% for FF samples diluted 1:10. The intra-assay variability for the progesterone ELISA assay was in the range from 3.6 to 8.9% and the inter-assay variability showed a range of 2.5 to 11.3% for FF samples diluted 1:2000. The data on the intra-assay variations for the 17-OH-progesterone ELISA assay was in the range from 5.7 to 10.6% for FF samples diluted 1:1000 during measurement. The inter-assay variability of this 17-OH-progesterone ELISA showed a range of 11.3 to 13.3% for FF samples diluted 1:1000 at testing.

The concentration of the steroid hormones in the FF samples was found stable at –20°C for 5–6 months since constant levels over time were observed with the five ELISA assays used.

Serum concentrations of estradiol were measured by Elecsys^®^ automated assay (Roche) whereas serum concentrations of all other steroid hormones were measured with validated LC-MS/MS methods on the day of oocyte recovery as reported previously (18).

### Gene expression of the LHR, *FSHR*, CYP19a1, and the androgen receptor in cumulus cells

RNA was isolated from cumulus cells (collected from one follicle per participant obtained during oocyte retrieval) using the Absolutely RNA Nanoprep Kit (Agilent Technologies, Catalog no.: 400753) according to the manufacturer’s instructions. The quality and quantity of the purified RNA were analysed with Bioanalyzer RNA 6000 Pico Kit (Agilent, SC, USA). Subsequently, cDNA was synthesized from the RNA with a high-capacity cDNA reverse transcription kit (Catalog # 4368814, Applied biosystems by Thermo Fisher Scientific) and used for quantitative real-time polymerase chain reaction (qRT-PCR). Predesigned TaqMan Gene Expression Assays for the following genes of interest were purchased from ThermoFisher scientific: *LHR* (id: Hs00174885_m1); *FSHR* (id: Hs01019695_m1); *CYP19a1* (id: Hs00903411_m1); *AR* (id: Hs00171172_m1) and TaqMan™ Fast Advanced Master Mix was applied (Catalog # 4444557, Applied Biosystems by Thermo Fisher Scientific). Gene expression levels were presented in relation to the housekeeping gene glyceraldehyde-3-phosphate dehydrogenase (GAPDH, id: Hs02786624_g1).

### Genotyping of LHR and *FSHR* SNPs in granulosa cells

GCs were isolated from the FF to assess SNPs in the LH and FSH receptors. The following SNPs were analyzed: rs2293275 (LHR 312, N312S) for *LHCGR* and rs6165 (*FSHR* 307, T307A) for *FSHR*. DNA was extracted from the GCs using the DNeasy Blood Kit (Qiagen, Hilden, Germany) according to the manufacturer’s protocol. Genotyping assays were performed to analyze the DNA samples for the SNPs of interest.

Genotyping was performed by PCR and high-resolution melting (HRM) analysis, using genotyping assays based on competitive amplification of differentially melting amplicons (CADMA), as previously described in detail (23) (**Supplementary Figure S1**). The CADMA method is based on a three-primer system including one reverse/forward primer designed to amplify the major allele only, and a second reverse/forward primer to amplify the minor allele only. Additionally, mismatches are included in the primers to either increase or decrease the melting temperature of the amplicon. The third common forward/reverse primer anneals to both sequences including the rare and the common allele. Thus, when PCR and HRM analysis are performed with the CADMA primers, the melting profile will reveal the genotype for each sample.

PCR and HRM analysis were performed on a LightCycler^®^480 Instrument II (Roche Diagnostics, Ropkreuz, Switzerland). The reaction mix included: 2 µL template DNA (10 ng/µL), 5 µL LightCycler^®^480 High Resolution Melting Master, 1.2 µL MgCl_2_ (25 µM), 0.3 µL N312S primer or 0.1 µL T307A primer specific for the minor allele (10µM), 0.1 µL N312S primer or 0.3 µL T307A primer specific for the major allele (10 µM), 0.2 µL common forward or reverse primer (10 µM) and 1.2 µL H_2_O to a final volume of 10 µL. Each sample was measured in duplicates. The melting profiles from the different patient samples were visualized and analyzed using the software function “Melt Curve Genotyping”.

We have designed and validated the two CADMA assays used in this study for detection of the SNPs of interest i.e., the rs2293275 (LHR 312, N312S) in the *LHCGR* and the rs6165 (*FSHR* 307, T307A). This was achieved by DNA sequencing where a 100% accordance was observed between the CADMA-based genotyping results and the DNA sequencing results. We have previously published the CADMA assay for detection of the rs6165 SNP in the *FSHR* gene (23), but this is the first time the CADMA assay detecting the rs2293275 SNP in the *LHCGR* gene is published, thus, validation data and HRM conditions are included in **Supplementary Table S1**.

### Statistical analysis

Steroid hormone concentrations and gene expression data were described and compared between the five CG beta doses and placebo using ANOVA models with age stratum (30–37 or 38–42 years) and AMH levels at screening (<15 pmol/L or ≥15 pmol/L) as factors. Multiplicative models were used, i.e., the concentrations were log-transformed before analysis. The results of the analyses were back-transformed and presented as estimated geometric means and mean treatment ratios with 95% confidence intervals and p-values. Values below the lower limit of quantification (LLOQ) were estimated with LLOQ/2.

For each of the different *FSHR* A307T SNPs and LHR N312S SNPs, patient characteristics and the number of oocytes retrieved were described. The dose-response relationships for steroid hormone concentrations and gene expression data were described using a linear model for the relationship between the dose of CG beta and the geometric mean in a log-normal distribution. Age stratum and AMH at screening were included as factors in the model. In an additional analysis, separate linear dose-response models were fitted for each of the different *FSHR* A307T SNPs and LHR N312S SNPs, and the p-value for the curves being SNP dependent was calculated.

## Results

### Serum FSH and LH levels at the start and end of ovarian stimulation

Following pituitary down-regulation, the overall endogenous FSH and LH levels were 3.25 (coefficient of variation [CV]=67%) IU/L and 1.49 (CV=71%) IU/L, respectively, as measured prior to the first gonadotropin injections. At the end of ovarian stimulation, serum FSH and LH levels were similar between the groups and overall, 14.8 (CV=63%) and 0.97 (CV=69%), respectively.

### Steroid hormone concentrations in follicular fluid

**Figure 1:**
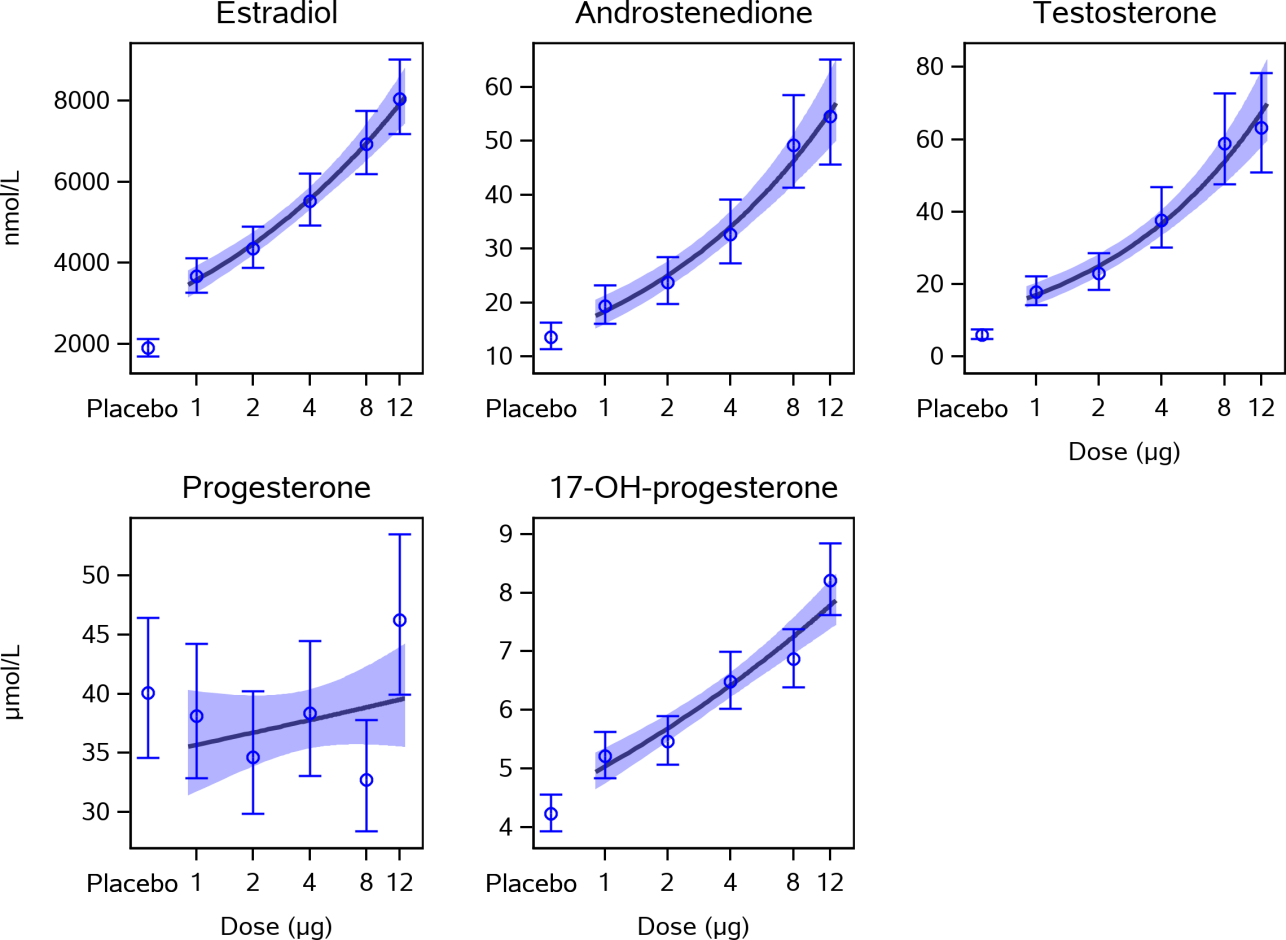
Dose-response effect of CG beta on intrafollicular levels of steroid hormones. This figure presents estimated means and 95% confidence intervals for steroid hormone concentrations measured in follicle fluid from human preovulatory follicles together with a linear approximation of the CG beta dose-response curve. The concentrations of estradiol, androstenedione, testosterone, and 17-OH-progesterone were all statistically significantly higher in all five CG beta dose groups when compared with placebo. The levels of progesterone did not differ significantly between the CG beta dose groups and placebo. P-values are shown in **Table 1**. CG, choriogonadotropin.

We found that CG beta has a pronounced positive dose-dependent effect on intrafollicular concentrations of 17-OH-progesterone, androstenedione, testosterone, and estradiol in human preovulatory follicles during OS with an individualized, fixed daily dose rFSH (**Table 1**). Compared with the placebo group, who received only rFSH stimulation without CG beta administration, the concentrations of steroids were dose-dependently increased, reaching in the highest dose group up to 2 times higher values for 17-OH-progesterone, up to 4 times higher values for estradiol and androstenedione, and up to 10 times higher values for testosterone without a clear plateauing (**Figure 1**). The concentrations of estradiol, androstenedione, testosterone, and 17-OH-progesterone were all significantly higher in all five CG beta dose groups when compared with placebo. However, for progesterone, there were no statistically significant differences between the CG beta dose groups and placebo.

**Table 1:** Statistical analysis of hormones in follicular fluid.

			Ratio vs Placebo		
Treatment	N	Mean	Estimate	95% Confidence Interval	P-value
Hormones in follicular fluids					
Estradiol (nmol/L)					
Placebo	93	1891			
1 µg	91	3664	1.94	(1.64; 2.28)	<.0001
2 µg	90	4352	2.30	(1.95; 2.71)	<.0001
4 µg	92	5523	2.92	(2.48; 3.44)	<.0001
8 µg	98	6929	3.66	(3.12; 4.30)	<.0001
12 µg	94	8047	4.26	(3.62; 5.01)	<.0001
Androstenedione (nmol/L)					
Placebo	93	13.64			
1 µg	91	19.35	1.42	(1.10; 1.83)	0.0072
2 µg	90	23.73	1.74	(1.35; 2.24)	<.0001
4 µg	92	32.69	2.40	(1.86; 3.09)	<.0001
8 µg	98	49.22	3.61	(2.81; 4.63)	<.0001
12 µg	94	54.55	4.00	(3.11; 5.15)	<.0001
Testosterone (nmol/L)					
Placebo	93	6.03			
1 µg	91	17.75	2.94	(2.16; 4.01)	<.0001
2 µg	90	22.91	3.80	(2.79; 5.18)	<.0001
4 µg	92	37.59	6.23	(4.58; 8.48)	<.0001
8 µg	98	58.91	9.77	(7.21; 13.23)	<.0001
12 µg	94	63.17	10.47	(7.71; 14.23)	<.0001
Progesterone (µmol/L)					
Placebo	93	40.09			
1 µg	91	38.13	0.95	(0.77; 1.17)	0.6394
2 µg	90	34.66	0.86	(0.70; 1.07)	0.1725
4 µg	92	38.34	0.96	(0.78; 1.18)	0.6753
8 µg	98	32.75	0.82	(0.67; 1.00)	0.0531
12 µg	94	46.22	1.15	(0.94; 1.42)	0.1777
17-OH-progesterone (µmol/L)					
Placebo	93	4.23			
1 µg	91	5.21	1.23	(1.11; 1.37)	0.0001
2 µg	90	5.46	1.29	(1.16; 1.44)	<.0001
4 µg	92	6.49	1.53	(1.38; 1.70)	<.0001
8 µg	98	6.86	1.62	(1.46; 1.80)	<.0001
12 µg	94	8.21	1.94	(1.75; 2.15)	<.0001

Multiplicative ANOVA model with treatment, age strata, and AMH group as factors. N, number of subjects with data; ANOVA, analysis of variance.

### Concentrations and relative increase in steroid hormones in serum versus follicle fluid

The concentration of steroid hormones in serum and in FF measured at oocyte retrieval as well as the relative increase with increasing dose of CG beta treatment are displayed in **Table 2**. The hormone concentrations were substantially higher in FF compared with serum and increasing doses of CG beta increased the levels of estradiol, 17-OH-progesterone, androstenedione, and testosterone in both serum and FF, although the increases in FF were larger, especially for testosterone. In contrast, CG beta treatment slightly decreased progesterone in circulation and did not affect progesterone concentrations in FF.

**Table 2:** Steroid hormone concentrations (nmol/L) in serum and follicle fluid and their relative increase upon CG beta treatment.

ENDPOINT	TREATMENT GROUP (hCG dose)						
		Placebo (0 µg)	1 µg	2 µg	4 µg	8 µg	12 µg
**Estradiol**	**Serum**	2.9 (100)	3.8^*^ (133)	4.3^*^ (147)	4.8^*^ (166)	6.2^*^ (215)	5.9^*^ (202)
	**FF**	1891 (100)	3664^*^ (194)	4352^*^ (230)	5523^*^ (292)	6929^*^ (366)	8047^*^ (426)
**Androstenedione**	**Serum**	6.2 (100)	8.3^*^ (134)	8.9^*^ (144)	10.4^*^ (169)	12.3^*^ (199)	11.9^*^ (194)
	**FF**	13.6 (100)	19.4^*^ (142)	23.7^*^ (174)	32.7^*^ (240)	49.2^*^ (361)	54.6^*^ (400)
**Testosterone**	**Serum**	1.7 (100)	2.3^*^ (136)	2.5^*^ (145)	2.9^*^ (171)	3.5^*^ (209)	3.3^*^ (196)
	**FF**	6.0 (100)	17.8^*^ (294)	22.9^*^ (380)	37.6^*^ (623)	58.9^*^ (977)	63.2^*^ (1047)
**Progesterone**	**Serum**	27.6 (100)	23.8 (86)	22.3^*^ (81)	19.5^*^ (71)	20.5^*^ (74)	19.6^*^ (71)
	**FF**	40090 (100)	38130 (95)	34660 (86)	38340 (96)	32750 (82)	46220 (115)
**17-OH-progesterone**	**Serum**	16.9 (100)	20.1^*^ (119)	22.1^*^ (131)	22.5^*^ (133)	26.8^*^ (158)	25.0^*^ (148)
	**FF**	4230 (100)	5210^*^ (123)	5460^*^ (129)	6490^*^ (153)	6860^*^ (162)	8210^*^ (194)

Mean values of steroid hormones (nmol/L) in serum and follicle fluid (FF) in relation to treatment group. The relative increase (%) of steroid hormone levels is indicated in parenthesis. *Denotes statistically significant difference compared with placebo (p<0.05). Results for FF and serum hormones are at oocyte retrieval. Steroid hormones were measured by different assays in serum and FF. hCG, human choriogonadotropin.

### Gene expression of the LH receptor, FSH receptor, CYP19a1 (aromatase), and the androgen receptor in cumulus cells

**Figure 2:**
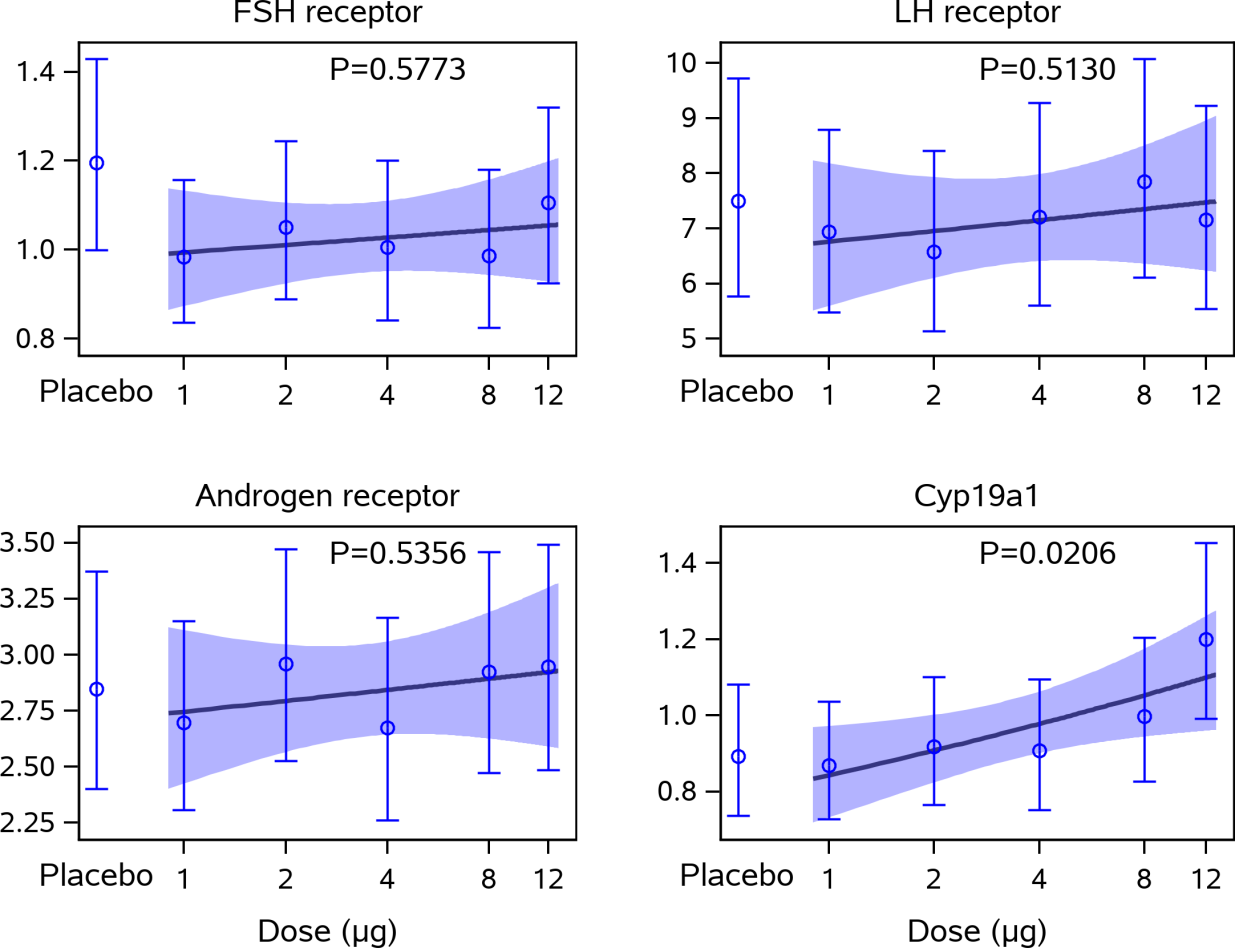
Gene expression levels in cumulus cells in relation to CG beta administration. This figure displays the estimated means and 95% confidence intervals for the relative gene expression levels of the FSHR (*FSHR*) x1000, LH Receptor (*LHCGR*) x1000, the Androgen Receptor (*AR*) x100, and *CYP19a1* to GAPDH together with a linear approximation of the CG beta dose-response curve. Statistical significance (multiplicative ANOVA model); *CYP19a1* expression in placebo versus 12 µg dose group: p = 0.0325. No other statistically significant differences were observed. ANOVA, analysis of variance; CG, choriogonadotropin; FSHR, follicle-stimulating hormone receptor; GAPDH (glyceraldehyde-3-phosphate dehydrogenase); LH, luteinising hormone.

The gene expression level of *CYP19a1* in the cumulus cells collected at oocyte retrieval increased significantly with increasing dose of CG beta (p=0.0206), mainly driven by the highest CG beta (12 μg) dose group (**Figure 2**). The gene expression of *CYP19a1* was significantly higher in the 12-µg dose group when compared with the placebo group (p=0.0325).

In contrast, the gene expression level of *FSHR* , *LHCGR* , and *AR* in cumulus cells were not affected by CG beta administration, as no dose-response trend with CG beta was observed.

### Distribution of *FSHR*/LHR genotypes in the study population

The frequencies of the different genotypes of the LHR N312S SNP (AA, AG, GG) and the *FSHR* A307T SNP (CC, CT, TT) are presented in **Supplementary Table S2**. Across the different treatment groups, 11.2–19.8% were homozygous for the A allele, 37.0–52.4% were heterozygous (AG), and 34.5–43.2% were homozygous for the G allele, in the LHR polymorphic site. For the *FSHR* SNP, 15.6–29.6% were homozygous for C allele, 42.0–52.3% were heterozygous (CT), and 28.4–35.8% were homozygous for the T allele.

### Number of oocytes retrieved in CG beta groups in relation to the *FSHR*/LHR genotypes

There was no clear overall difference in the number of oocytes retrieved between the different *FSHR* (307) or LHR (312) genotypes (**Supplementary Tables S3**, **S4**).

### Intrafollicular steroid hormone concentrations in relation to the *FSHR*/LHR genotypes according to hCG dose group

**Figure 3:**
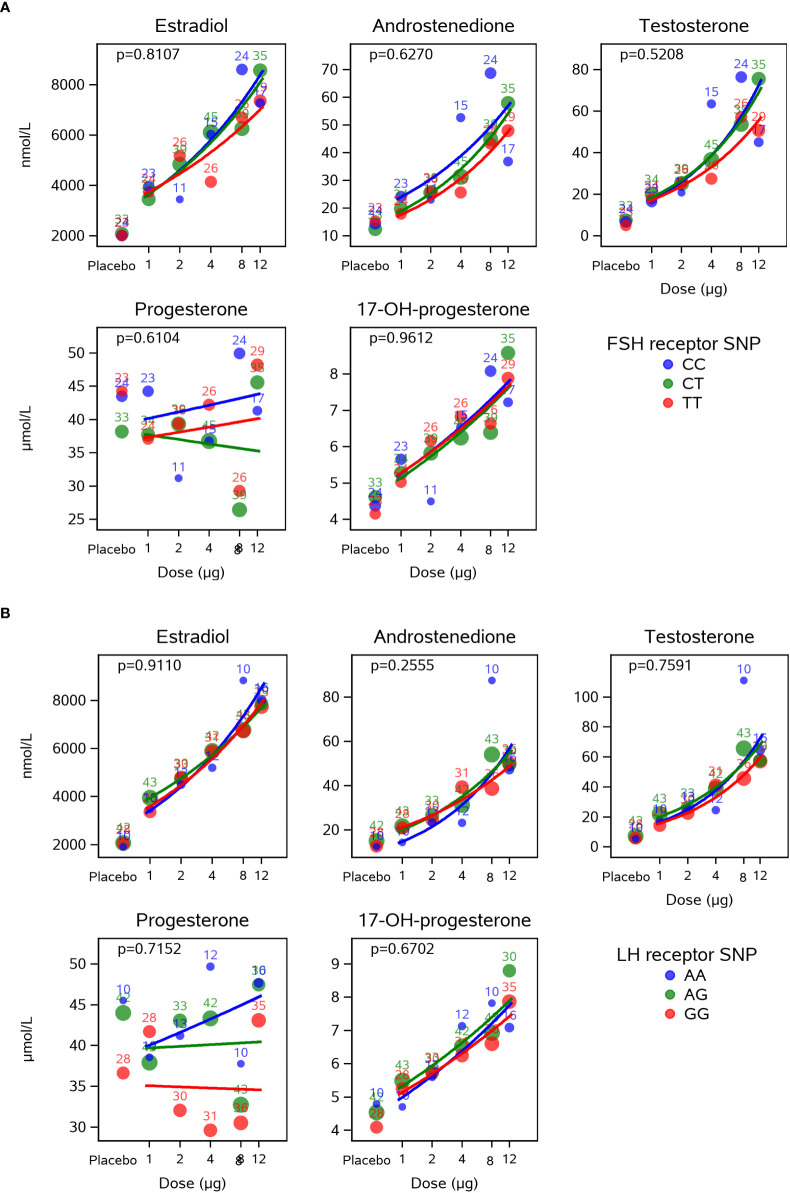
Dose-response effect of CG beta on intrafollicular levels of steroid hormones is not influence by the LHR N312S or the FSHR A307T SNP genotypes. This figure presents estimated CG beta dose-response curves for each of the FSHR A307T **(A)** and the LHR N312S **(B)** SNP genotypes. Bubbles indicate mean values for each dose with the size of the bubble indicating the number of subjects (also included as numbers). The dose-response curves did not depend on the genotypes of the analysed SNPs for any of the five steroid hormones. CG, choriogonadotropin; LHR, luteinising hormone receptor; FSHR, follicle-stimulating hormone receptor; SNP, single nucleotide polymorphism.

No significant differences were observed between the CG beta dose-response curves for steroid hormone levels when comparing the different *FSHR* A307T SNPs and LHR N312S SNPs (**Figure 3**). Thus, the increase concentrations observed for estradiol, androstenedione, testosterone, and 17-OH-progesterone in response to increasing CG beta levels were not influenced by the genotypes of the LHR N312S SNP or the *FSHR* A307T SNP.

## Discussion

The patients included in this study received a long GnRH agonist protocol of daily subcutaneous triptorelin that secured a strong pituitary suppression of endogenous LH and FSH. Following this protocol, serum LH concentrations were on average 1 IU/L during OS, thus, steroidogenesis was driven only by rhFSH in patients without CG beta treatment (the placebo group), whereas in patients treated with CG beta, steroidogenesis was effectively augmented in a dose-dependent manner demonstrating the importance of LH-like activity during stimulation. The daily doses of CG beta were relatively high, ranging from 1 to 12 µg resulting in steady-state levels of 0.1 to 1.0 ng/mL (18).

This analysis demonstrates a clear positive correlation in the intrafollicular steroid hormone concentrations, except for progesterone, as a response to increasing dose of CG beta during OS with an individualized, fixed daily dose of rhFSH (follitropin delta). The intrafollicular concentrations increased between two and ten times compared with the control group (placebo), which were stimulated with rhFSH only. This clearly shows that OS, with both FSH and LH-like activity, largely affects steroid levels in the FF in which maturing oocytes are exposed up to the moment of their retrieval. It is noted that on the day of oocyte retrieval, increases of steroid hormones in FF due to treatment with CG beta in comparison to placebo treatment were much higher than those measured in the circulation (18). Furthermore, none of the evaluated steroids showed signs of reaching a plateau with the highest concentrations of CG beta administered, which suggests that the GC output had not yet reached a ceiling. This high output of steroids may reflect conditions of OS, where the supraphysiological concentrations of FSH throughout the follicular phase are likely to result in greater stimulation of *LHCGR* expression on GCs as compared with the natural menstrual cycle and thereby creates highly responsive GCs that readily undertake steroidogenesis (2). The studies by Loumaye and colleagues (8) reported an LH-induced ceiling effect regarding follicular growth in WHO group I and II anovulatory women undergoing OS for ovulation induction. Indeed, this intervention trial also showed that multiple follicle growth was attenuated due to too high CG beta exposure (18), but that the ceiling of follicular steroidogenesis was not reached. This was further supported by the relative increase in sex-steroid concentrations in serum and FF.

In the current study, a CG beta dose-dependent increase of progesterone was not observed at the day of oocyte retrieval, neither in serum nor in FF. This observation was also reported by Thuesen and co-workers (24) who, in a similar long GnRH agonist protocol, administered a daily dose of 50, 100 or 150 IU urinary hCG during OS with recombinant FSH (24–26). However, in Thuesen’s study, FF concentrations of testosterone, androstenedione, and estradiol reached a plateau at a urinary hCG daily dose of 100 IU whereas in our study these steroids increased to much higher concentrations beyond this plateau and maximum levels were still not reached at a daily dose of CG beta 12 µg, indicating a relatively high potency. Taken together, during OS where supraphysiological levels of FSH are likely to secure a high *LHCGR* expression on GCs, CG beta exerts a strong dose-dependent stimulation of ovarian steroidogenesis with no apparent upper ceiling reached in normal responder patients, which may be related to the unique glycosylation and longer elimination half-life of CG beta in comparison to other hCG preparations (17).

The absence of CG beta-induced progesterone at the day of oocyte recovery suggests that the mechanism controlling progesterone production may be different from those acting on the production of downstream steroids. In agreement with this, Loumaye and colleagues previously reported an increase in estradiol secretion when rLH was co-administered with FSH, whereas progesterone levels remained unchanged (8). This may be related to the fact that 17-OH-progesterone is the immediate metabolite downstream of progesterone catalyzed by 17α-hydroxylase, 17,20 lyase enzyme (gene: *CYP17a1*) in the Δ4 pathway of ovarian steroidogenesis. The human orthologue of 17α-hydroxylase catalyzes the conversion of 17-OH-progesterone to androstenedione only to a very limited extent in the Δ4 pathway and because 3β-hydroxysteroid dehydrogenase (HSD) has no back conversion from Δ4 to Δ5 metabolites, 17-OH-progesterone is a terminal product in human ovarian steroidogenesis (27, 28). In contrast, the Δ5 pathway is readily open wherein the 17α-hydroxylase converts 17-OH-pregnenolone to dehydroepiandrosterone (DHEA), which *via* 3β-HSD is a precursor for androstenedione and later testosterone. 17-OH-progesterone showed a similar pattern of increase as androstenedione, testosterone, and estradiol contrasting that of progesterone. There is no obvious explanation for this difference, but it is hypothesized that the 17α-hydroxylase is strongly upregulated securing a very effective conversion of progesterone to 17-OH-progesterone in the presence of CG beta along with an increase in the androgen production and thus subsequently production of estradiol. Even though the action of 17α-hydroxylase takes place outside the follicle, in the theca cells, it is nonetheless likely to be reflected in the FF. Further *in vitro* studies using isolated theca cells exposed to different concentrations of CG beta would help elucidate mechanisms that control production of downstream steroids.

Another explanation could be that the affinity for pregnenolone towards 3β-HSD (converting Δ5 steroids to Δ4 steroids) may be reduced compared with 17-OH-pregnenolone. This may cause an attenuated conversion of pregnenolone to progesterone and an enhanced conversion of 17-OH-pregnenolone to 17-OH-progesterone during the conditions created by the OS protocol used in this study.

The gene expression of *LHCGR* , *FSHR* , *CYP19a1*, and *AR* is tightly regulated during the ovulatory peak. We found a significant upregulation of *CYP19a1* gene expression by CG beta of high dose in cumulus cells after induction of final maturation of follicles. This upregulation corresponds well with the observed upregulation of estradiol in the corresponding FF. CG beta did not seem to have an impact on the expression of *LHCGR* , *FSHR* , or *AR* at the time of oocyte retrieval, since no difference was observed between the different treatment groups.

The expression of gonadotropin receptors is strongly affected by the mid-cycle surge of gonadotropins with a strong initial downregulation that, as the ovulatory process progresses, slowly start to recover close to ovulation (29, 30). It is, therefore, a dynamic period of gonadotropin receptor expression, which the huge variability of our current results probably reflects.

It is important to note that there exist significant functional differences between cumulus cells and mural GCs, and the *LHCGR* expression on these cell types differs. Cumulus cells were used for the gene expression analysis in this study and since the *LHCGR* is much more abundantly expressed in mural GCs (31, 32), a different result may have been obtained for these cells.

One of the limitations of the current study is that only one FF-sample was collected per patient, which, however, is counteracted by the fact that the study comprised data from 558 patients. Furthermore, data reflect the luteinized follicle, thus interference by CG beta prior to final maturation of follicles may have been affected by the hCG trigger.

The two SNPs selected for analysis in this study were rs2293275 (LHR N312S) in the *LHCGR* gene and rs6165 (*FSHR* A307T) in the *FSHR* gene. These SNPs are among the most studied polymorphic sites of the *LHCGR* gene and *FSHR* gene, respectively (19–23). The LHR N312S is located near a glycosylation site and there are studies indicating that variations in the sequence could affect the *LHCGR* sensitivity (21). Similarly, there are studies indicating that *FSHR* polymorphisms may have an impact on the sensitivity and function of the *FSHR* (19, 20). More recent publications also indicate that *LHCGR* and *FSHR* polymorphisms may have an impact on the chance of pregnancy as well as the cumulative live birth rate after *in vitro* fertilization treatment (21, 22). In the current study, neither polymorphism of the *FSHR* or *LHCGR* affected the ovarian response which was for each subset aligned with the participant’s ovarian reserve. This finding is in good agreement with another study of rhFSH (follitropin delta) showing that three *FSHR* SNPs (including *FSHR* A307T) had no predicting value in terms of ovarian response and gonadotropin efficiency (33) and with an earlier study that demonstrated that the *FSHR* A307T SNP did not affect the peak estradiol levels, number of preovulatory follicles, and number of retrieved oocytes, although there was a difference in the number of FSH ampoules needed for successful stimulation (19). In addition, with respect to the intrafollicular steroid hormone levels, no clear difference was observed between the different CG beta dose groups based on the SNP variants, suggesting that, in this trial population, the effect of CG beta on intrafollicular steroid hormones was not affected by the different *FSHR* and *LHCGR* genotypes.

In conclusion, in a large group of women with profound pituitary suppression in a long GnRH agonist protocol, the addition of CG beta during OS with an individualized, fixed dose of rFSH was shown to increase steroid output significantly and dose-dependently, except for progesterone. This study clearly demonstrates the pronounced effect that LH-like activity exerts on steroidogenesis following OS. In this context, in which the pre-ovulatory follicles are exposed to supraphysiological concentrations of rFSH, there was no apparent ceiling effect of CG beta on steroidogenesis as measured directly in the FF.

## Data availability statement

The original contributions presented in the study are included in the article/**Supplementary Material**. Further inquiries can be directed to the corresponding author.

## Ethics statement

The studies involving human participants were reviewed and approved by Commissie Medische Ethiek, Laarbeeklaan 101, 1090 Brussels, Belgium; Etické komise pro multicentrické klinické hodnocení Fakultní nemocnice v Motole V Úvalu 84, 150 06 Praha 5, Czech Republic; De Videnskabsetiske Komiteer for Region Hovedstaden, Regionsgården, Kongens Vænge 2, 3400 Hillerød, Denmark; CEIm Provincial de Sevilla, Hospital Universitario Virgen Macarena de Sevilla, Avenida Doctor Fedriani 3, Sevilla, 41009, Spain; West of Scotland Research Ethics Service, Clinical Research and Development, West Glasgow Ambulatory Care Hospital, Dalnair Street, Glasgow G3 8SW, United Kingdom; NHS GCC, Clinical Research and Development, West Glasgow Ambulatory Care Hospital, Dalnair Street, Glasgow G3 8SJ, United Kingdom; and HRA and Health and Care Research Wales (HCRW), Castle Bridge 4, 15-19 Cowbridge Rd E, Cardiff CF11 9AB, United Kingdom. The patients/participants provided their written informed consent to participate in this study.

## Author contributions

CA, SK, and BM provided substantial contribution to the trial design. PL performed the statistical analysis. JB performed the genotyping analysis and drafted the manuscript. HO performed the gene expression analysis. All authors contributed to the data interpretation, reviewed multiple versions of the manuscript and approved the final version.

## Funding

The study was funded by Ferring Pharmaceuticals, Copenhagen, Denmark.

## Acknowledgments

The authors would like to thank all investigators and their medical staff of the Rainbow trial (18) for collecting follicular fluid and cumulus cells, as well as the women who consented and donated samples for the study. We would also like thank Marjo Westerdahl for her technical assistance with the ELISA measurements and validation. Finally, we would like to thank Ida Lindgren from Lund University in Sweden for performing DNA sequencing to validate our CADMA primers. University in Sweden for performing DNA sequencing to validate our CADMA primers.

## Conflict of interest

BM and PL are employees of Ferring Pharmaceuticals. CA has received payment for lectures from IBSA, Switzerland, and his laboratory was reimbursed for the analyses performed under this study.

The remaining authors declare that the research was conducted in the absence of any commercial or financial relationships that could be construed as a potential conflict of interest.

The authors declare that this study received funding from Ferring Pharmaceuticals. The funder was involved in the study design, collection, analysis, interpretation of data, the writing of this article and the decision to submit it for publication

## Publisher’s note

All claims expressed in this article are solely those of the authors and do not necessarily represent those of their affiliated organizations, or those of the publisher, the editors and the reviewers. Any product that may be evaluated in this article, or claim that may be made by its manufacturer, is not guaranteed or endorsed by the publisher.
