# Corrigendum: Dose-dependent stimulation of human follicular steroidogenesis by a novel rhCG during ovarian stimulation with fixed rFSH dosing

**DOI:** 10.3389/fendo.2024.1397017

**Published:** 2024-03-21

**Authors:** Jane Alrø Bøtkjær, Stine Gry Kristensen, Hanna Ørnes Olesen, Per Larsson, Bernadette Mannaerts, Claus Yding Andersen

**Affiliations:** ^1^ Laboratory of Reproductive Biology, University Hospital of Copenhagen, Rigshospitalet, Copenhagen, Denmark; ^2^ Global Biometrics, Ferring Pharmaceuticals A/S, Copenhagen, Denmark; ^3^ Reproductive Medicine & Maternal Health, Ferring Pharmaceuticals A/S, Copenhagen, Denmark; ^4^ Faculty of Health and Medical Sciences, University of Copenhagen, Copenhagen, Denmark

**Keywords:** Steroidogenesis, FE 999302, recombinant hCG, ovarian stimulation, cumulus cells, single nucleotide polymorphisms

In the published article, there was an error in Table 2 as published. The incorrect data was included in the last row of the table for 17-OH-progesterone as these were a duplicate of the testosterone values from a few rows above. The corrected table in this corrigendum (**Table 1**) and its caption Steroid hormone concentrations (nmol/L) in serum and follicle fluid and their relative increase upon CG beta treatment appear below on the next page.

**Table 1:** Steroid hormone concentrations (nmol/L) in serum and follicle fluid and their relative increase upon CG beta treatment.

	TREATMENT GROUP (hCG dose)						
ENDPOINT		Placebo (0 µg)	1 µg	2 µg	4 µg	8 µg	12 µg
**Estradiol**	**Serum**	2.9 (100)	3.8^*^ (133)	4.3^*^ (147)	4.8^*^ (166)	6.2^*^ (215)	5.9^*^ (202)
	**FF**	1891 (100)	3664^*^ (194)	4352^*^ (230)	5523^*^ (292)	6929^*^ (366)	8047^*^ (426)
**Androstenedione**	**Serum**	6.2 (100)	8.3^*^ (134)	8.9^*^ (144)	10.4^*^ (169)	12.3^*^ (199)	11.9^*^ (194)
	**FF**	13.6 (100)	19.4^*^ (142)	23.7^*^ (174)	32.7^*^ (240)	49.2^*^ (361)	54.6^*^ (400)
**Testosterone**	**Serum**	1.7 (100)	2.3^*^ (136)	2.5^*^ (145)	2.9^*^ (171)	3.5^*^ (209)	3.3^*^ (196)
	**FF**	6.0 (100)	17.8^*^ (294)	22.9^*^ (380)	37.6^*^ (623)	58.9^*^ (977)	63.2^*^ (1047)
**Progesterone**	**Serum**	27.6 (100)	23.8 (86)	22.3^*^ (81)	19.5^*^ (71)	20.5^*^ (74)	19.6^*^ (71)
	**FF**	40090 (100)	38130 (95)	34660 (86)	38340 (96)	32750 (82)	46220 (115)
**17-OH-progesterone**	**Serum**	16.9 (100)	20.1^*^ (119)	22.1^*^ (131)	22.5^*^ (133)	26.8^*^ (158)	25.0^*^ (148)
	**FF**	4230 (100)	5210^*^ (123)	5460^*^ (129)	6490^*^ (153)	6860^*^ (162)	8210^*^ (194)

Mean values of steroid hormones (nmol/L) in serum and follicle fluid (FF) in relation to treatment group. The relative increase (%) of steroid hormone levels is indicated in parenthesis. *Denotes statistically significant difference compared with placebo (p<0.05). Results for FF and serum hormones are at oocyte retrieval. Steroid hormones were measured by different assays in serum and FF. hCG, human choriogonadotropin.

The authors sincerely apologize for this error and state that this does not change the scientific conclusions of the article in any way. The original article has been updated.

