# Supplementary material for: Dose-dependent stimulation of human follicular steroidogenesis by a novel rhCG during ovarian stimulation with fixed rFSH dosing
Source: Front Endocrinol (Lausanne). 2022 Oct 20;13:1004596. doi: 10.3389/fendo.2022.1004596 (PMC9632659; doi:10.3389/fendo.2022.1004596)

Supplementary Figure S1. Validated CADMA genotyping assay for the LHCGR variant N312S (rs2293275). A. The sequence of the LHCGR N312S CADMA primers in colored arrows and the corresponding gene sequence of the LHCGR around the SNP of interest (**G/A**). The first primer (blue arrow) is designed to amplify the N312 variant (**A**), with two melting temperature (temp) decreasing mutations introduced. The second primer (green arrow) is designed as a common forward primer. The third primer (red arrow) is designed to amplify the 312S variant (**G**), with two melting temperature increasing mutations introduced. B. Melting curves and peaks obtained after optimised HRM conditions using the CADMA primers. The three melting profiles identify the genotypes of three patients run in duplicates showing the homozygous GG in red, the heterozygous GA in purple, and the homozygous AA in blue. The amplicons differ in nucleotide content according to the genotype, and this is reflected in the melting temperatures. C. Three chromatograms showing results from DNA sequencing of three patients with different LHCGR genotypes defined with the CADMA genotyping assay, thus confirming, and validating the CADMA primers.

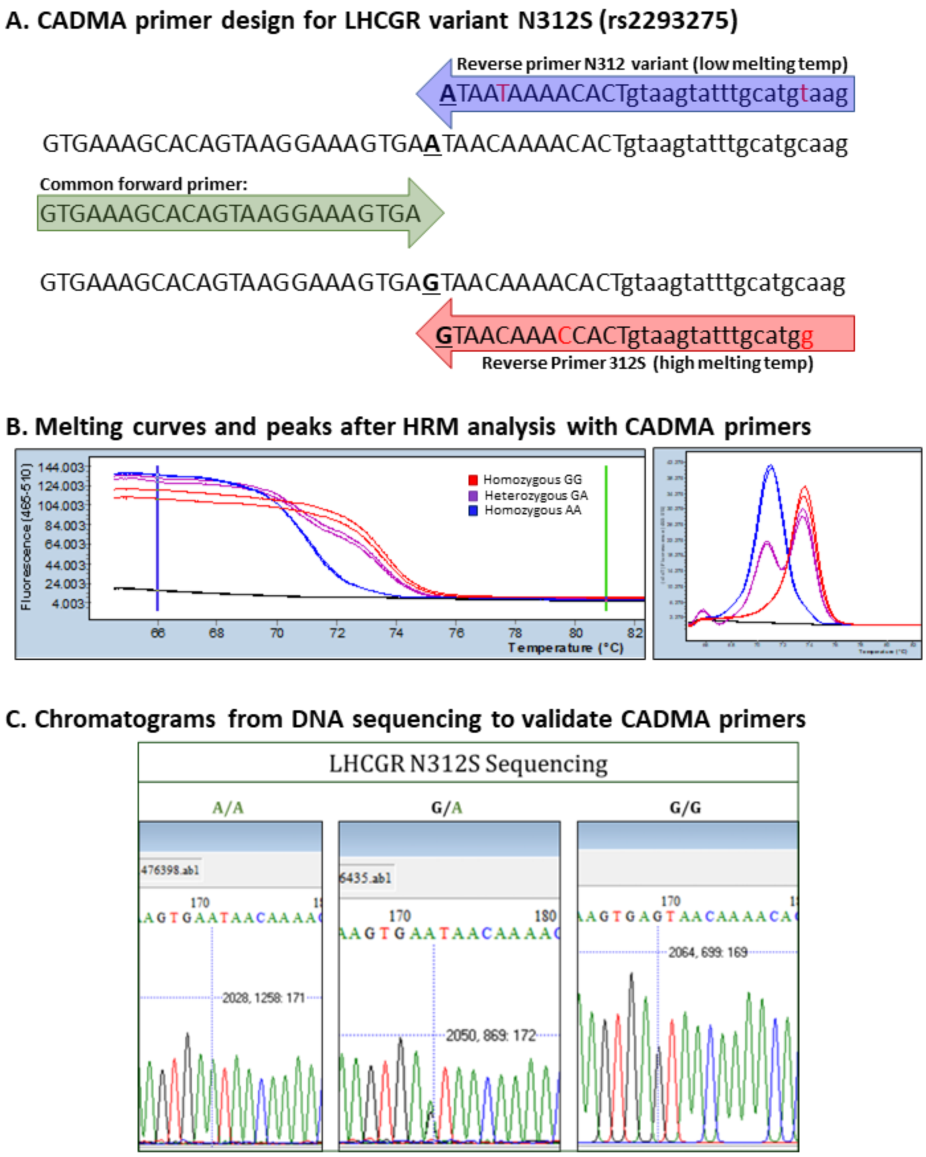

Supplement: Supplementary file 1 [file Image_1.pdf]
