# Supplementary material for: Dose-dependent stimulation of human follicular steroidogenesis by a novel rhCG during ovarian stimulation with fixed rFSH dosing
Source: Front Endocrinol (Lausanne). 2022 Oct 20;13:1004596. doi: 10.3389/fendo.2022.1004596 (PMC9632659; doi:10.3389/fendo.2022.1004596)
Supplement: Supplementary file 2 [file Table_1.docx]

**Supplementary Table S1. PCR and HRM program settings for analysis of the N312S LHCGR variant**

| Program name | Cycles | Program details | Analysis Mode |
| --- | --- | --- | --- |
| Preincubation | 1 | 95 C°, 5 min, 4.4 C°/s | None |
| Amplification | 45 | 95 C°, 10 sec, 4.4 C°/s | Quantification Mode |
|  |  | 51 C°, 15 sec, 2.2 C°/s |  |
|  |  | 70 C°, 10 sec, 4.4 C°/s |  |
| Melting | 1 | 95 C°, 1 min, 4.4 C°/s | Melting curves |
|  |  | 65 C°, 1 min, 2.2 C°/s |  |
|  |  | 65 C°, 1 min, 4.4 C°/s |  |
|  |  | 95 C°, -, 0.01 C°/s |  |

LHCGR, luteinising hormone/human chorionic gonadotrophin receptors; HRM, high resolution melting; PCR, polymerase chain reaction.
