# Supplementary material for: Dose-dependent stimulation of human follicular steroidogenesis by a novel rhCG during ovarian stimulation with fixed rFSH dosing
Source: Front Endocrinol (Lausanne). 2022 Oct 20;13:1004596. doi: 10.3389/fendo.2022.1004596 (PMC9632659; doi:10.3389/fendo.2022.1004596)
Supplement: Supplementary file 3 [file Table_2.docx]

**Supplementary Table S2. The distribution of the FSHR/LHR genotypes in relation to treatment group**

____________________________________________________________________________________________________

Placebo 1 ug 2 ug 4 ug 8 ug 12 ug

(N=104) (N=104) (N=101) (N=99) (N=107) (N=104)

____________________________________________________________________________________________________

LH receptor SNP, n (%)

AA 10 (12.3) 11 (13.1) 13 (16.9) 12 (14.1) 10 (11.2) 16 (19.8)

AG 42 (51.9) 44 (52.4) 33 (42.9) 42 (49.4) 43 (48.3) 30 (37.0)

GG 29 (35.8) 29 (34.5) 31 (40.3) 31 (36.5) 36 (40.4) 35 (43.2)

All 81 (100.0) 84 (100.0) 77 (100.0) 85 (100.0) 89 (100.0) 81 (100.0)

FSH receptor SNP, n (%)

CC 24 (29.6) 23 (27.4) 12 (15.6) 15 (17.4) 24 (27.0) 17 (21.0)

CT 34 (42.0) 36 (42.9) 39 (50.6) 45 (52.3) 39 (43.8) 35 (43.2)

TT 23 (28.4) 25 (29.8) 26 (33.8) 26 (30.2) 26 (29.2) 29 (35.8)

All 81 (100.0) 84 (100.0) 77 (100.0) 86 (100.0) 89 (100.0) 81 (100.0)

_________________________________________________________________________

FSHR, follicle-stimulating hormone; LHR, luteinising hormone.
