# Supplementary material for: Dose-dependent stimulation of human follicular steroidogenesis by a novel rhCG during ovarian stimulation with fixed rFSH dosing
Source: Front Endocrinol (Lausanne). 2022 Oct 20;13:1004596. doi: 10.3389/fendo.2022.1004596 (PMC9632659; doi:10.3389/fendo.2022.1004596)
Supplement: Supplementary file 4 [file Table_3.docx]

**Supplementary Table S3. Summary statistics by FSH A307T receptor SNP**

|  | **CC (N=115)** | **CT (N=228)** | **TT (N=155)** | |
| --- | --- | --- | --- | --- |
| ***Age (years)*** | 35.3 ± 3.21 | 35.6 ± 3.40 | 35.5 ± 3.42 | |
| ***AMH (pmol/L)*** | 15.6 ± 6.74 | 14.9 ± 7.14 | 15.7 ± 6.73 | |
| ***Weight (kg)*** | 66.1 ± 11.4 | 66.1 ± 10.8 | 65.0 ± 10.3 | |
| ***Antral follicle count*** | 14.1 ± 6.00 | 12.8 ± 5.14 | 13.9 ± 6.00 | |
| ***Oocytes retrieved*** | 11.4 ±5.25 | 11.1 ± 5.02 | 11.5 ± 5.64 | |
| Data are mean ± standard deviation. | | | |  |

FHS, Follicle stimulating hormone; SNP, single nucleotide polymorphism.
