# Supplementary material for: Dose-dependent stimulation of human follicular steroidogenesis by a novel rhCG during ovarian stimulation with fixed rFSH dosing
Source: Front Endocrinol (Lausanne). 2022 Oct 20;13:1004596. doi: 10.3389/fendo.2022.1004596 (PMC9632659; doi:10.3389/fendo.2022.1004596)
Supplement: Supplementary file 5 [file Table_4.docx]

**Supplementary Table S4. Summary statistics by LH N312S receptor SNP**

|  | **AA (N=72)** | **AG (N=234)** | **GG (N=191)** |
| --- | --- | --- | --- |
| ***Age (years)*** | 35.3 ± 3.33 | 35.5 ± 3.38 | 35.6 ± 3.47 |
| ***AMH (pmol/L)*** | 15.8 ± 7.51 | 15.6 ± 6.72 | 14.9 ± 6.95 |
| ***Weight (kg)*** | 67.1 ± 10.4 | 65.1 ± 10.7 | 66.0 ± 10.9 |
| ***Antral follicle count*** | 14.6 ± 6.72 | 13.5 ± 5.36 | 12.9 ± 5.49 |
| ***Oocytes retrieved*** | 12.5 ± 5.54 | 11.6 ± 5.28 | 10.5 ± 5.06 |
| Data are mean ± standard deviation. | | | |
